# How similar is the antibacterial activity of silver nanoparticles coated with different capping agents?†

**DOI:** 10.1039/d3ra00917c

**Published:** 2023-04-03

**Authors:** Ana M. Ferreira, Anna Vikulina, Michael Loughlin, Dmitry Volodkin

**Affiliations:** a School of Science and Technology, Department of Chemistry and Forensics, Nottingham Trent University Clifton Lane Nottingham NG11 8NS UK dmitry.volodkin@ntu.ac.uk; b Bavarian Polymer Institute, Friedrich-Alexander-Universität Erlangen-Nürnberg (FAU) Dr-Mack-Straße, 77 90762 Fürth Germany

## Abstract

Silver nanoparticles (AgNPs) represent one of the most commercialised metal nanomaterials, with an extensive number of applications that span from antimicrobial products to electronics. Bare AgNPs are very susceptible to aggregation, and capping agents are required for their protection and stabilisation. The capping agents can endow new characteristics which can either improve or deteriorate AgNPs (bio)activity. In the present work, five different capping agents were studied as stabilizing agents for AgNPs: trisodium citrate (citrate), polyvinylpyrrolidone (PVP), dextran (Dex), diethylaminoethyl-dextran (Dex^DEAE^) and carboxymethyl-dextran (Dex^CM^). The properties of the AgNPs were studied using a set of methods, including transmission electron microscopy, X-ray diffraction, thermogravimetric analysis and ultraviolet-visible and infrared spectroscopy. Coated and bare AgNPs were also tested against *Escherichia coli*, methicillin-resistance *Staphylococcus aureus* and *Pseudomonas aeruginosa* to analyse their capacity to suppress bacterial growth and eradicate biofilms of clinically relevant bacteria. The results showed that all the capping agents endow long-term stability for the AgNPs in water; however, when the AgNPs are in bacterial culture media, their stability is highly dependent on the capping agent properties due to the presence of electrolytes and charged macromolecules such as proteins. The results also showed that the capping agents have a substantial impact on the antibacterial activity of the AgNPs. The AgNPs coated with the Dex and Dex^CM^ were the most effective against the three strains, due to their better stability which resulted in the release of more silver ions, better interactions with the bacteria and diffusion into the biofilms. It is hypothesized that the antibacterial activity of capped AgNPs is governed by a balance between the AgNPs stability and their ability to release silver ions. Strong adsorption of capping agents like PVP on the AgNPs endows higher colloidal stability in culture media; however, it can decrease the rate of Ag^+^ release from the AgNPs and reduce the antibacterial performance. Overall, this work presents a comparative study between different capping agents on the properties and antibacterial activity of AgNPs, highlighting the importance of the capping agent in their stability and bioactivity.

## Abstract 2

Nanosilver antibacterial activity is governed by a balance between the nanosilver stability and their ability to release/dope Ag^+^ to solution.

## Graphical abstract

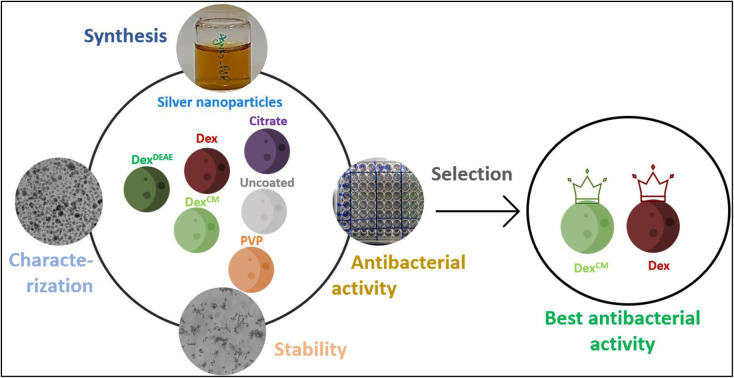

## 1. Introduction

Silver nanoparticles (AgNPs) are one of the most commercialised nanomaterials due to their attractive physical–chemical and biological properties, being found in a diverse number of applications, such as antimicrobial products and electronics.^1^ AgNPs can be produced through different routes: chemical, physical and biological, with the chemical route being one the most commonly used.^1,2^ In the chemical reduction method, AgNPs are synthesised by reducing ionic silver with a reducing agent, like sodium borohydride or sodium citrate. The synthesis of AgNPs through this route is quick, cost-effective and simple, with the properties of the nanoparticles, including size, being adjusted with additives, reagent ratios, synthesis temperature, pH and stirring speed.^3,4^ Despite the simplicity of this route, tight control is required to prevent the overgrowth of the nanoparticles. Within seconds, the nanoparticles can transform into large sub-micron particles or form nanoparticle aggregates. To help control the growth of the AgNPs, and assure long-term stability, capping agents are used. These agents are usually added during synthesis and adsorb to the surface of nanoparticles, endowing stability *via* decreasing the surface energy and promoting repulsion forces (electrostatic, van der Waals or hydration) or steric hindrance.^5^ The repulsive forces can co-exist with steric hindrance, and both can prevent the over-growth and agglomeration between adjacent particles.^5,6^ Numerous capping agents are applied as stabilisers for AgNPs, with polyvinylpyrrolidone (PVP) and trisodium citrate (citrate) being two of the most used ones, due to their good stabilisation properties and safety.^7^ Dextran (Dex) and the derivatised forms diethylaminoethyl-dextran (Dex^DEAE^) and carboxymethyl-dextran (Dex^CM^) have also been used as capping agents, although to a much lower extent than PVP and citrate.^8–16^

The selection of the capping agent is crucial to assure that the AgNPs do not agglomerate when exposed to destabilisers, like electrolytes, and lose their unique properties and biocidal activity. The antimicrobial activity of AgNPs is size-dependent. Smaller nanoparticles present better antibacterial activity, as they release more silver ions (Ag^+^), and more easily interact and penetrate the cell membranes.^17,18^ The toxicity mechanisms of AgNPs aren't fully understood but it is believed that the release of Ag^+^ from the nanoparticles is crucial to kill the bacteria. Ag^+^ can disrupt the membrane, and once uptaken by the cell, interact with disulfide or sulfhydryl groups of intracellular enzymes leading to the disruption of metabolic processes like adenosine triphosphate release, and increasing the production of reactive oxygen species.^2,17^ Alongside that, Ag^+^ can interact with the DNA, affecting DNA replication and cell propagation, and denature the cytoplasmic ribosomal components hindering protein synthesis.^2,17^ It is believed that the AgNPs also play a role in killing the bacteria through denaturation of the membrane and modification of the cell wall structure, which can lead to leakage of cellular contents and cell death.^2,17^ Nonetheless, Xiu *et al.*,^18^ have shown that the antibacterial activity of AgNPs against *Escherichia coli* (*E. coli*) mainly relies on Ag^+^ release, highlighting the role of the ions over the particles in eradicating the bacteria.

Based on the antibacterial mechanisms of the AgNPs, the capping agents play an important role in assuring and potentiating the antibacterial activity, mainly by preventing the formation of clusters, but also by endowing new characteristics that can change the interactions with the bacteria and release rate of Ag^+^.

In this work, AgNPs were synthesised and stabilised with five different capping agents: citrate, PVP, Dex, Dex^DEAE^ and Dex^CM^. Bare nanoparticles were also synthesised for comparison. The produced AgNPs were characterised through various techniques, to investigate the effect of the capping agents on the physicochemical properties of the AgNPs, as well as on the stability in different media. The antibacterial activity of the AgNPs was assessed against *E. coli*, methicillin-resistant *Staphylococcus aureus* (MRSA) and *Pseudomonas aeruginosa* (*P. aeruginosa*) to study how the capping agents influence the antibacterial activity.

Overall, this work presents a comparative study where the role of the capping agents, their properties and sensitivity to external factors are investigated in an attempt to highlight the importance of a holistic approach in the design of stable AgNPs.

## 2. Results and discussion

### 2.1. AgNPs characterization

#### 2.1.1. Effect of the capping agents on the optical properties of the AgNPs and synthesis reproducibility

The production of AgNPs *via* the chemical route requires the use of capping agents to prevent the growth over nanoscale dimensions and endow stability by preventing their agglomeration and formation of large clusters of nanoparticles. The capping agents stabilise the nanoparticles *via* adsorption on their surface, decreasing the surface energy and promoting repulsion forces and steric hindrance.^5,6^ Numerous capping agents are used as stabilisers for AgNPs, including dextrans, PVP and trisodium citrate, with the last two being the most common ones.^7^ The selection of the capping agent is an important step, as it affects the properties, stability and consequently the final activity of the nanoparticles.

In this work PVP, citrate and Dex^DEAE^ were chosen as capping agents to stabilise the AgNPs. The concentration of citrate was set to 0.2 mg mL^−1^ based on the work of Izak-Nau E. *et al.*^19^ and the PVP and Dex^DEAE^ final concentrations were selected based on preliminary studies (Fig. S1†), where AgNPs were synthesised with different concentrations of PVP and Dex^DEAE^ and then analysed by UV-vis spectroscopy. This method is a good option for analysing AgNPs as it is highly sensitive to their size, shape and polydispersity.^20^ Spherical AgNPs with sizes around 10 nm, and with low polydispersity, tend to present a narrow peak with maximum absorbance in the 400 nm region, due to the surface plasmon resonance of the AgNPs.^20^ As shown in Fig. S1,† increasing the PVP concentration from 0.09 to 0.38 mg mL^−1^ resulted in AgNPs with lower polydispersity, as indicated by the narrower peaks. This can be explained by the increment of the surface coverage with PVP, which increased the stability of the AgNPs through steric hindrance *via* its bulky structure and repulsive forces created by the hydrophobic carbon chains.^7^ At concentrations above 0.38 mg mL^−1^ no changes were verified in the spectra, which indicates the saturation of the AgNPs with PVP. Interestingly, the AgNPs synthesised with Dex^DEAE^ required lower concentrations of Dex^DEAE^ to stabilise the AgNPs (0.13 mg mL^−1^), indicating a good affinity between Dex^DEAE^ and the AgNPs. Based on the results presented in Fig. S1,† the PVP and Dex^DEAE^ concentrations were set at 0.38 and 0.13 mg mL^−1^, respectively, as at these ratios, the AgNPs presented narrow peaks with maximum absorbance around 400 nm.

**Fig. 1:**
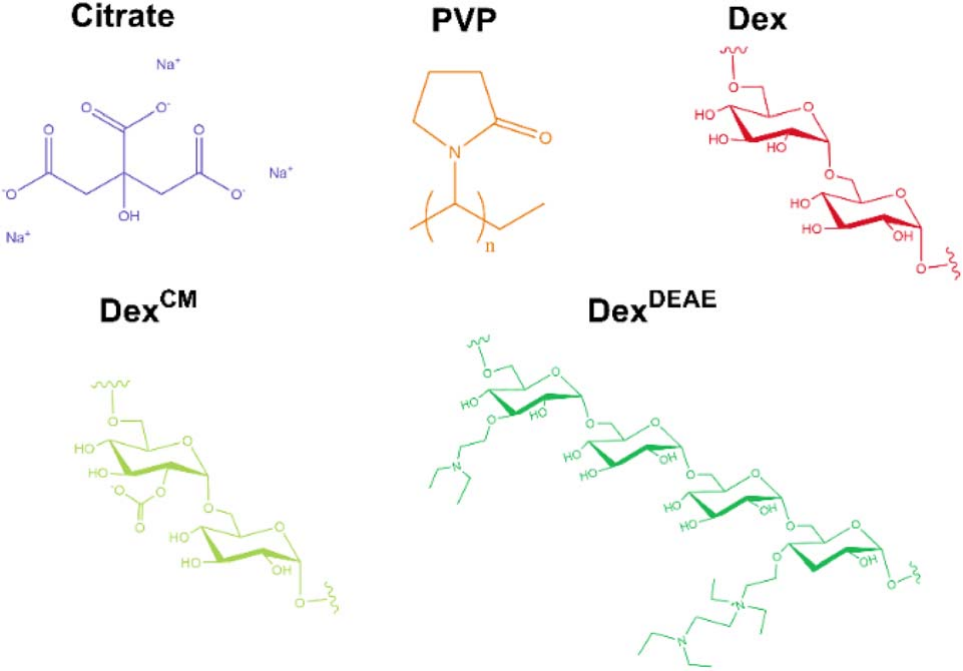
Chemical structure of the capping agents used to stabilise the AgNPs.

Two other dextrans with the same molecular weight (40k), Dex and Dex^CM^, were also tested as stabilising agents to further evaluate the effect of dextrans charge on the properties of the AgNPs. The concentration of Dex and Dex^CM^ was also set at 0.013 mg mL^−1^, as at this concentration stable AgNPs with a narrow size distribution were formed (Fig. S2†). The chemical structures of the capping agents are presented in Fig. 1.

**Fig. 2:**
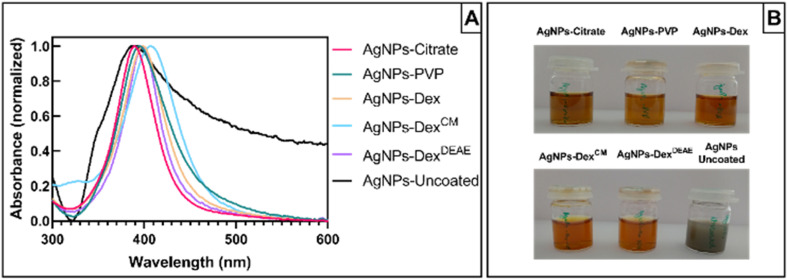
UV-vis absorbance spectra of the bare AgNPs (AgNPs-uncoated) and the AgNPs coated with different capping agents after washing (A), and the colloidal dispersions of all AgNPs after synthesis (B).

Fig. 2A presents the UV-vis absorbance spectra of the AgNPs synthesised without any capping agent (AgNPs-uncoated) and with citrate (AgNPs-citrate), PVP (AgNPs-PVP), Dex (AgNPs-Dex), Dex^CM^ (AgNPs-Dex^CM^), and Dex^DEAE^ (AgNPs-Dex^DEAE^) as stabilisers. All the AgNPs presented maximum absorbance between 385 and 406 nm (Table S1†), proving the formation of AgNPs. While the AgNPs synthesised with capping agents presented narrow peaks and colloidal dispersions with a characteristic amber colour (Fig. 2B), the uncoated AgNPs had a broad absorbance peak, distinctive of highly polydisperse colloids, and presented a greyish colour, an indicator of nanoparticles with bigger sizes.

To demonstrate the reproducibility of the AgNPs production between synthesis, three independent batches of AgNPs were analysed by UV-vis spectroscopy. As presented in Fig. S2,† the absorbance spectra between the batches of the coated AgNPs are very similar, with the maximum absorbance peak around 400 nm demonstrating the reproducibility of the synthesis method and the formation of AgNPs with sizes around 10 nm. The AgNPs synthesised without stabiliser also presented similar absorbance spectra between batches, although with some differences, showing that the reproducibility of AgNPs synthesis without capping agents is harder to achieve.

#### 2.1.2. Size distribution

The size of the AgNPs is one of the most important characteristics as it drastically affects the antibacterial activity, with smaller nanoparticles being associated with better antimicrobial activity.^17^

**Fig. 3:**
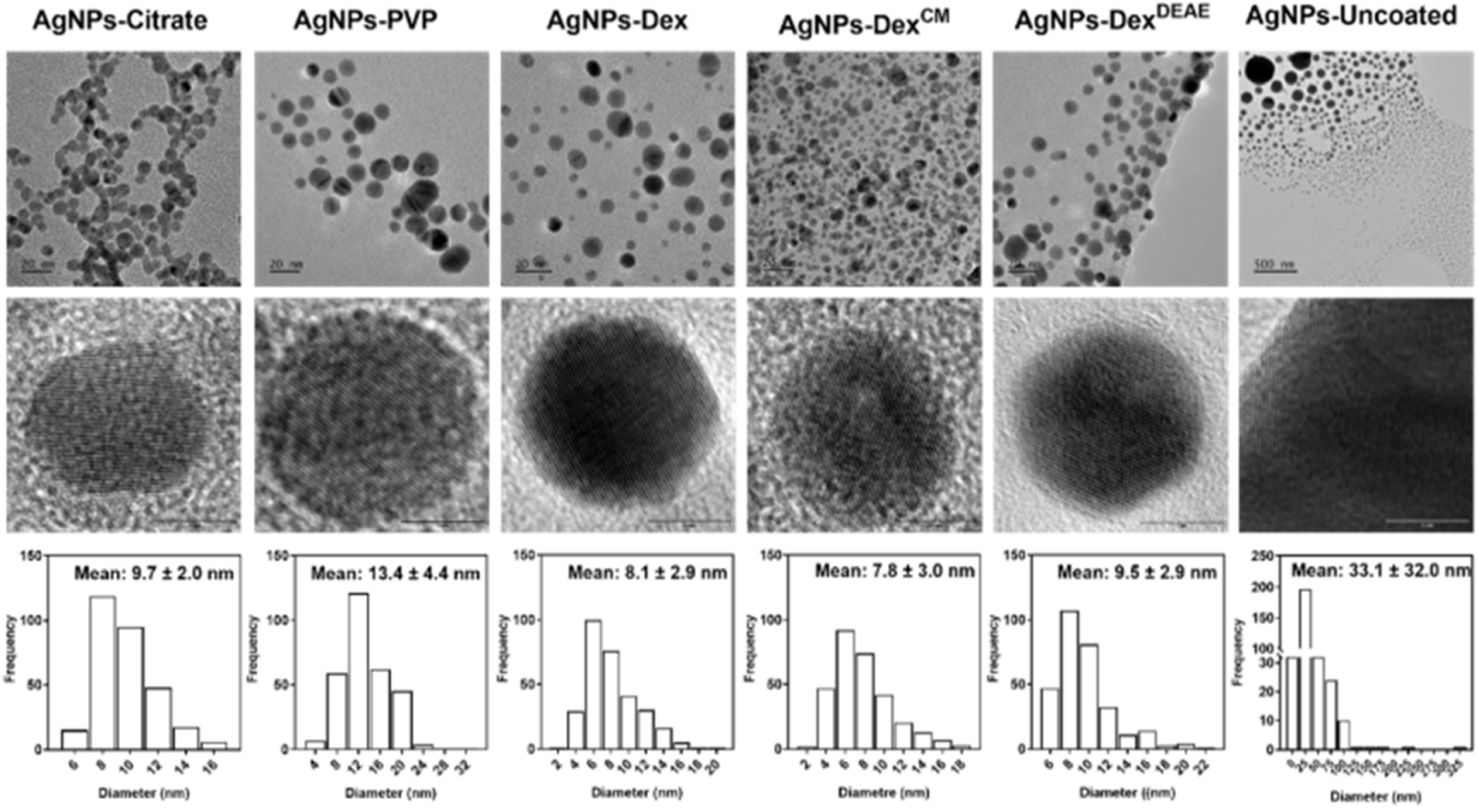
TEM images of the synthesised AgNPs and corresponding size distribution histograms.

The size of the synthesised nanoparticles was studied by TEM and DLS. Fig. 3 depicts the TEM images and size distribution histograms of all the AgNPs. The AgNPs-citrate, AgNPs-PVP, AgNPs-Dex, AgNPs-Dex^CM^, AgNPs-Dex^DEAE^ and AgNPs-uncoated presented the following average diameters determined by TEM: 9.7 ± 2.0; 13 ± 4.4; 8.1 ± 2.9, 7.8 ± 3.0; 9.5 ± 2.9 and 33.1 ± 32.0 nm, respectively. The data shows that all the nanoparticles coated with a stabilising agent presented an average size around 10 nm with a Gaussian-like distribution. On the other hand, the uncoated AgNPs presented larger sizes and a broad distribution, as shown by the size distribution histogram and TEM images, where it is possible to see particles with diameters of 10 and 300 nm.

While TEM mainly gives information about the diameter of the inorganic core of metallic nanoparticles, as it is more sensitive to electron-dense regions, DLS provides information about the hydrodynamic size of the nanoparticles, which corresponds to the inorganic core, capping agent and adsorbed solvent layer.^21,22^ Fig. S3† presents the hydrodynamic diameter of the nanoparticles measured by DLS (intensity weighted). All the AgNPs presented two size clusters, one with maximum intensity around 10 nm and the other at 70 nm, except for the AgNPs coated with Dex^CM^, which presented maximum intensity for the first and second peaks around 22 and 140 nm, respectively. This data shows that the hydrodynamic diameter of the AgNPs present a bimodal distribution, with the average size determined by TEM coinciding with the first peak. The second peak, around 70–140 nm, can be explained by four different reasons: (1) the fact that DLS reflects the hydrodynamic diameter; (2) the AgNPs not being perfectly monodisperse; (3) the lower accuracy of DLS analysis for bimodal distributions, and (4) the distortion effect of larger nanoparticles on DLS results, as the diameter of larger nanoparticles is heavily weighted, and therefore, even if larger nanoparticles are present in lower numbers they will have much higher intensities due to the intensity of scattered light being proportional to the sixth power of the radius.^23,24^ Interestingly, DLS analysis did not detect the large nanoparticles found by TEM on the uncoated AgNPs colloidal dispersions, which can be explained by the sedimentation of these nanoparticles/clusters during DLS analysis.

#### 2.1.3. Crystalline structure

**Fig. 4:**
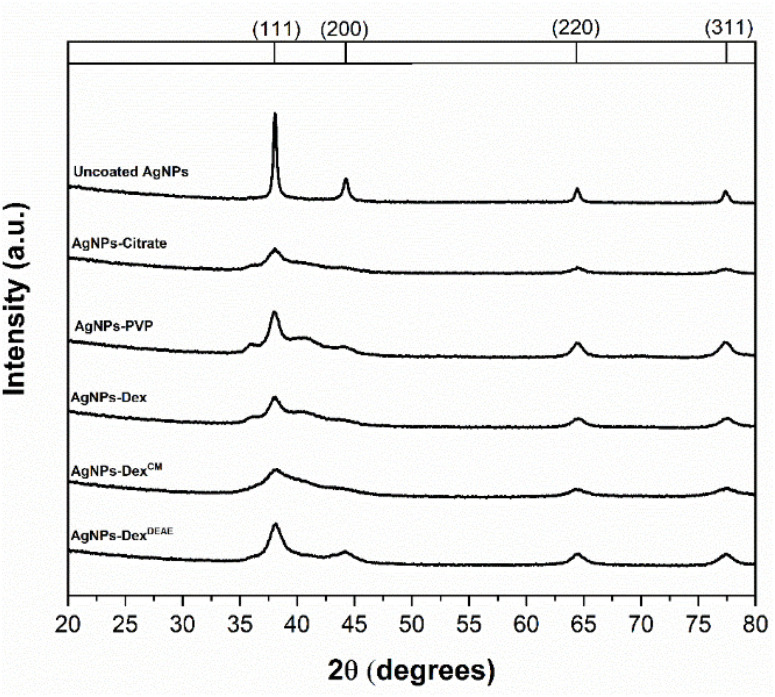
X-ray diffraction pattern of the AgNPs.

The crystalline structure of the AgNPs was studied by powder XRD. Fig. 4 depicts the diffraction pattern of all the produced AgNPs. Regardless of the type of capping agent, or absence of stabiliser, all the AgNPs presented a face-centred cubic crystal structure with the characteristic peaks of silver metallic nanoparticles around 38, 44, 64 and 77°, assigned to the crystal planes (111), (200), (220) and (311), respectively (card no. 9008459, Rigaku database). The crystalline structure of the AgNPs is also evidenced in the TEM images (Fig. 3, middle row), where it is possible to see the lattices in all AgNPs. The absence of the main silver oxide crystals peaks on the diffraction pattern at 32.17, 37.31, 53.79, 64.08 and 67.29° (card no. 7109246, Rigaku database) also indicates that the nanoparticles correspond to pure silver.

Two of the major differences between the XRD pattern of the uncoated AgNPs and the stabilised ones are the peak intensity and broadening. The uncoated AgNPs presented more intense and narrow peaks than the stabilised AgNPs, where in some cases, some of the peaks are difficult to discern. This is due to the size of the nanoparticles. The smaller the AgNPs, the higher the peak broadening and the lower the signal-to-noise ratio.^25^ These results are in agreement with the UV-vis and TEM data, which show that the uncoated nanoparticle colloids present particles with larger diameters.

#### 2.1.4. Surface charge and chemistry

The stabilisation of nanoparticles with capping agents implies the alteration of the surface chemistry to create repulsion forces and steric hindrance that prevents the agglomeration of the particles upon collision with one another. Table 1 presents the zeta potential of the AgNPs in Milli-Q water which is consistent with our previous study.^26^ All the nanoparticles, except for the AgNPs-Dex^DEAE^, had a negative zeta potential (−21.9 to −36.4 mV) which is due to the polyanionic nature of some of the capping agents, *i.e.*, citrate ions and Dex^CM^, and the presence of electrons on the surface of metallic nanoparticles which are sufficient to endow a negative charge due to the low mass of the AgNPs.^27,28^ The AgNPs coated with Dex^DEAE^, were the only nanoparticles that presented a positive potential (*ca.* 45 mV), due to the positively charged diethylaminoethyl moieties on the derivatised dextran.

**Table 1:** Zeta potential of the AgNPs in Milli-Q water. The values correspond to the average of 50 runs

AgNPs	Zeta potential (mV)
AgNPs-citrate	−30.6 ± 5.3
AgNPs-PVP	−24.0 ± 8.0
AgNPs-Dex	−21.9 ± 7.0
AgNPs-Dex^CM^	−36.4 ± 6.0
AgNPs-Dex^DEAE^	45.2 ± 6.9
AgNPs-uncoated	−32.5 ± 9.9

The charge on the surface of the nanoparticles helps to promote their stability through repulsion forces. Allied with that, the stability of the coated AgNPs is increased by steric hindrance promoted by the bulky structures of the capping agents.

Fig. S4† presents the infrared spectra of the coated AgNPs and the pure capping agents (citrate, PVP, Dex, Dex^CM^ and Dex^DEAE^). The data shows that the capping agents are responsible for the spectral signatures of the nanoparticles. All the AgNPs present characteristic peaks of the capping agent, with shifts in the wavelength being present due to the interactions with the AgNPs surface. In the case of the AgNPs coated with citrate, the nanoparticles presented bands with strong intensities at 1553 and 1376 cm^−1^, which correspond to the asymmetric and symmetric stretching vibration of the carboxylate groups, respectively.^29,30^

The AgNPs coated with PVP presented the characteristic bands of PVP, with an intense band at 1660 cm^−1^ resulting from C=O stretching, and two other bands at 1290 and 1020 cm^−1^ resulting from C–N stretching. The redshifts of these bands demonstrate the interaction of PVP with silver, as these interactions occur through the carbonyl group and nitrogen atom of the pyrrolidone ring.^7^ It has been reported that PVP also interacts with silver through van der Waals attraction and direct binding, which explains the redshift and different intensity of the bands attributed to C–H bending at 1490–1420 cm^−1^.^7^

All the AgNPs coated with dextran, or derivatised dextran, presented similar spectra due to their chemical similarity. The AgNPs presented the characteristic bands resultant from the glycosidic bonds at 1149–1151 cm^−1^, 1021–1024 cm^−1^ and 916–918 cm^−1^.^31,32^ Interestingly, the spectra of the AgNPs coated with dextrans presented a more evident band at *ca.* 1040 cm^−1^. This band has been associated with a more-ordered structure,^32^ and seems to indicate that the dextrans adsorbed on the surface of the AgNPs present a well-organised structure. The AgNPs coated with dextrans also presented more intense bands at *ca.* 1419–1386 cm^−1^, assigned to in-plane bending of the C–H bond,^33^ and seem to result from the interaction of the dextrans with the nanoparticles.

Overall, the data shows that the capping agents adsorbed on the surface of the nanoparticles, resulting in AgNPs with different charges and spectral identities.

#### 2.1.5. Capping agent content

**Fig. 5:**
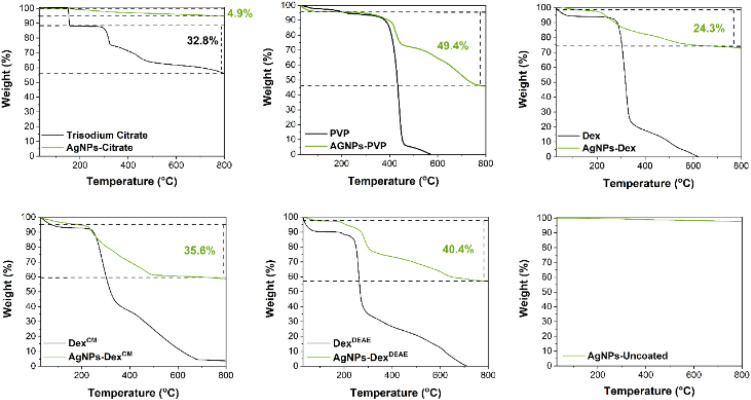
TGA curve of the AgNPs and corresponding capping agents.

Thermogravimetric analysis was carried out to study the thermal stability of the AgNPs and the content of the capping agent adsorbed on the nanoparticles. Uncoated AgNPs and pure capping agents were used as controls. As depicted in Fig. 5, all the coated AgNPs present a weight variation below 5% between 30 and 200 °C, resultant from the evaporation of the water adsorbed on the nanoparticles. All the capping agents showed an almost complete degradation at 600–700 °C, except for citrate. Regarding the amount of capping agent adsorbed on the nanoparticles, this ranged between *ca.* 5 and 50%. The percentage of citrate ions adsorbed on the surface of the nanoparticles is predicted to be above 4.9% and below 14.9%. The last value was determined considering that the weight loss (4.9%) accounted for 32.8% of the total content of the capping agent. This value is overestimated, as the capping agent corresponds to only citrate ions provided by the dissolution of the trisodium citrate dihydrate salt. Nonetheless, this estimation helps to determine a percentage range of the citrate content adsorbed on the nanoparticles. The AgNPs-PVP presented a high content of adsorbed PVP (49.4%) on its surface, corresponding to almost 50% of the total mass of the nanoparticles. The AgNPs-Dex, AgNPs-Dex^CM^ and AgNPs-Dex^DEAE^ presented the following percentages of adsorbed capping agent: 24.3, 35.6 and 40.4%, respectively. The different percentages demonstrate that the dextrans have different affinities to the nanoparticles, with Dex and Dex^DEAE^ presenting the lowest and highest affinity, respectively. These results show that the diethylaminoethyl and carboxymethyl moieties on the derivatised dextran, increase the affinity to the nanoparticles, possibly by creating more interactions with the surface. This data also explains why lower concentrations of dextran solutions (0.13 mg mL^−1^, or below) in comparison with PVP (0.38 mg mL^−1^, or above) resulted in stable AgNPs even when stabilised with Dex. The high affinity between the dextrans and the AgNPs increased their adsorption, with low concentrations being enough to form a stabilising layer.

The different content of capping agents on the AgNPs is likely to be reflected in the hydrodynamic diameter data determined by DLS (Fig. S3†). The AgNPs with lower contents of capping agent (AgNPs-citrate and AgNPs-Dex) presented slightly more narrow distributions (lower polydispersity index) with the peak centred around 10 nm being more intense in comparison to the AgNPs coated with PVP, Dex^CM^ and Dex^DEAE^.

### 2.2. Stability of the AgNPs

The antibacterial and optical properties of AgNPs are size-dependent, and therefore it is of utmost importance to monitor their size during storage and application. In the next sections, the stability of AgNPs after storage, and the effect of different media on their properties, are studied.

#### 2.2.1. Storage stability

The colloidal stability of the AgNPs, when stored at 4 °C for 9 months, was assessed to study the capacity of the different capping agents to maintain the colloidal stability of the nanoparticles. Fig. S5A† presents the UV-vis spectra of the AgNPs, with and without coating, before (0 months) and after being stored for 9 months (9 months). The UV-vis is a good method to monitor the stability of the AgNPs, as the peak around 400 nm is sensitive to the size of the nanoparticles. The data shows that the size of the AgNPs did not change substantially over time, as the UV-vis spectra are identical before and after storage.

The images of the AgNPs dispersions after storage (Fig. S5B†) show that the coated AgNPs kept their colloidal stability as there was no detectable sedimentation of the nanoparticles on the bottom of the flask. On the other hand, the uncoated AgNPs presented the formation of a deposit, highlighting their limited colloidal stability and the importance of the capping agents to prolong the stability of the nanoparticles.

#### 2.2.2. Stability in bacterial growth media

The culture media used to perform the antibacterial tests present rich compositions to provide all the nutrients required by the bacteria. Their composition varies according to the type of medium and can include starch, casein hydrolysate, peptone, meat infusions, meat/yeast extracts, and salts. While these components promote optimal growth, some adsorb on the surface of the nanoparticles forming a corona that changes the biological identity of the nanoparticles.^34^ This corona can help to increase the stability of the nanoparticles but also changes their activity.^35^ Other components present in culture media, like salts and charged biomolecules, can also disrupt the stability of the nanoparticles and promote the formation of clusters, reducing the total surface area, and consequently the antibacterial activity.^35^ Moreover, chloride salts, like NaCl, which are found in a vast number of media, can either decrease or increase the activity of AgNPs by reacting with free Ag^+^ forming insoluble precipitates of AgCl or soluble silver chloride complexes AgCl_*x*_^(*x*−1)−^. The formation of AgCl or AgCl_*x*_^(*x*−1)−^ complexes is dependent on the concentration of Cl^−^, with higher concentrations favouring the formation of silver chloride complexes, which increases the toxicity of the AgNPs.^36^ Due to the complexity of the culture media, and the myriad of formulations existent in the market containing components that can affect the stability, surface charge, release and availability of Ag^+^, comparisons between antibacterial tests reported by different research groups are challenging.^35^

Fig. S6† and 6 present the UV-vis spectra and transmittance images of the AgNPs in different culture media and PBS before and after incubation at 37 °C, respectively. The composition of the culture media and PBS are presented in Table S2,† and the images of the nanoparticles dispersions before and after incubation, are present in Fig. S7,† as the colour change of the dispersions is a quick and reliable indicator of the AgNPs stability.

**Fig. 6:**
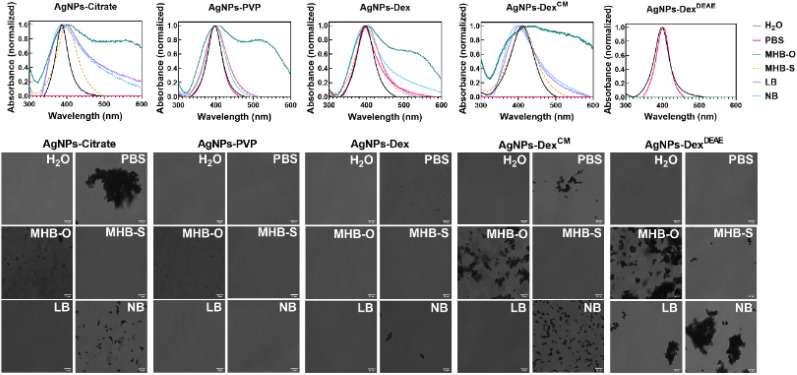
UV-vis spectra and respective transmittance images of the AgNPs in deionised water (H_2_O), PBS and different broths used for bacterial growth: MHB-O, MHB-S, LB and NB after mixing and overnight incubation at 37 °C.

Overall, the data shows that the AgNPs stability varies with the type of media, with the AgNPs coated with PVP being the most stable nanoparticles across all the media tested. Nonetheless, after overnight incubation at 37 °C, micro-sized clusters of AgNPs-PVP formed in MHB-O, as demonstrated in Fig. S6.† The AgNPs coated with Dex^DEAE^ were extremely unstable in all the media tested, which was enhanced during incubation at 37 °C. As shown in Fig. S6 and S7,† the maximum absorbance band around 400 nm disappeared due to the formation of large silver clusters and the nanoparticle flocculation. On the other hand, the AgNPs-Dex^DEAE^ presented good stability in PBS, demonstrating that the instability in the culture media was triggered by the interaction of the biomolecules in the media with Dex^DEAE^, promoting its displacement from the surface of the AgNPs.

The AgNPs coated with citrate, Dex, and Dex^CM^ showed some degree of instability in PBS, with the AgNPs coated with citrate and Dex^CM^ immediately clustering and losing their surface plasmon resonance properties (Fig. 6). As shown in Fig. S7,† the colour of the nanoparticle dispersions in PBS changed from amber to grey and then became transparent due to the flocculation of the nanoparticles. This was caused by the salts present in PBS, which destabilised the electric repulsions created by the citrate ions and Dex^CM^ negative charges, triggering the irreversible agglomeration of the nanoparticles. Another mechanism of destabilization may be the competition of phosphate ions, also negatively charged, with citrate ions and Dex^CM^ for adsorbing on the surface of the AgNPs. Phosphate ions can replace in part or completely the capping agents and reduce the colloidal stability of the AgNPs by forming a less organized diffuse layer of ions.

All the nanoparticles after incubation presented some degree of instability in MHB-O, which can be explained by the high content of dehydrated infusion from meat (300 g L^−1^). On the other hand, MHB-S was the media that less disturbed the stability of the AgNPs, and for that reason, was selected for the antibacterial tests.

Overall, the data shows that the AgNPs are highly sensitive to the composition of the media and buffers, with positively charged nanoparticles being extremely unstable in culture media. The higher the content of electrolytes and meat infusion or extracts, the higher the chance of disrupting the stability of the nanoparticles, with the nanoparticles stabilised through electrostatic repulsions being more susceptible.

### 2.3. Antibacterial activity of the AgNPs

#### 2.3.1. MIC and MBC

The antibacterial activity of the AgNPs was assessed against *E. coli*, MRSA and *P. aeruginosa*, three strains responsible for a high number of healthcare associated infections.^37,38^

**Fig. 7:**
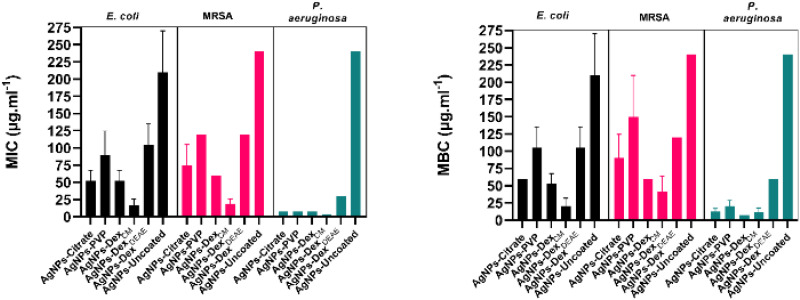
Minimum inhibitory concentration (MIC) and minimum biocidal concentration (MBC) of AgNPs. The concentration represents the content of silver in the nanoparticles. The medium used was MHB-S. The experiment was repeated three times, with three replicates per repetition.

Fig. 7 presents the average MIC and MBC of all the AgNPs. The average MIC ranged between 16.9–210 μg mL^−1^, 18.8–240 μg mL^−1^ and 3.8–240 μg mL^−1^ for *E. coli*, MRSA and *P. aeruginosa*, respectively. The wide ranges demonstrate that the MIC varied considerably between the AgNPs. The uncoated AgNPs were the nanoparticles that presented the lowest activity, caused by the large nanoparticles and broad size distribution. Moreover, the lack of a capping agent made the bare AgNPs more susceptible to agglomeration in the medium. The AgNPs capped with Dex^DEAE^ presented the second-highest MIC. This is explained by their instability in the medium, which promoted their agglomeration as demonstrated in the previous section. The formation of clusters is very detrimental to the activity of AgNPs, as the total surface area is reduced, decreasing the interactions with the bacteria and the release of Ag^+^, essential for the antibacterial activity.^2^

Despite the good stability of the AgNPs-PVP in MHB-S, their antibacterial activity was lower than the activity of the AgNPs coated with citrate, Dex or Dex^CM^. This can be explained by the slightly larger diameter (13.4 ± 4.4 nm) of the AgNPs-PVP in comparison with the AgNPs-citrate (9.7 ± 2.0 nm), AgNPs-Dex (8.1 ± 2.9 nm) and AgNPs-Dex^CM^ (7.8 ± 3.0 nm). Additionally, PVP itself, and the corona formed on the nanoparticles through the adsorption of biomolecules from the medium, might also play a role, as they can decrease the interactions with the bacteria and the release of Ag^+^. It is also expectable that PVP forms a tight mesh on the surface of the nanoparticles due to the hydrophobic nature of the carbon chain, decreasing the oxidation of the AgNPs and consequent release of Ag^+^. On the other hand, the hydrophilic nature of the dextrans and citrate results in a loose protective layer that allows a quicker diffusion of oxygen and Ag^+^. Interestingly, the AgNPs-PVP and AgNPs Dex^DEAE^ presented similar MICs against *E. coli* and MRSA, despite the AgNPs-Dex^DEAE^ being considerably more unstable. This might be due to the positively charged AgNPs-Dex^DEAE^ presenting stronger interactions with the negatively charged bacterial cell surfaces^39^ and to a higher oxidation rate that result in the release of more Ag^+^. The AgNPs coated with citrate, Dex and Dex^CM^ presented the lowest MIC values, with the AgNPs-Dex^CM^ presenting the best antibacterial activity against *E. coli* and MRSA, due to being more stable in MHB-S, as shown in Fig. 6 and S6,† than the other nanoparticles. Nonetheless, other factors might have also potentiated the antibacterial activity of the AgNPs-Dex^CM^, like the type of corona formed on their surface, and the release rate of Ag^+^.

The MBC data presented the same trend found in the MIC results, although higher concentrations were needed to kill the bacteria. The average MBC varied between 20–210 μg mL^−1^, 41–240 μg mL^−1^ and 11–240 μg mL^−1^ for *E. coli*, MRSA and *P. aeruginosa*, respectively. The MIC and MBC of the coated AgNPs are similar to values reported by other authors.^40–43^ Nonetheless, a significant discrepancy between studies in the literature is found due to variations in the AgNPs properties (size, zeta potential, shape, capping agent) and the assay conditions (inoculum density, medium formulation and salts concentration).

To study the effect of the capping agents on the antibacterial results, all the capping agents were tested against the same bacteria at concentrations above their content in the AgNPs dispersions with biocidal activity. As depicted in Fig. S8,† the bacteria grew in the presence of the capping agents (cloudy wells), ruling out their bactericidal activity at the concentrations tested. The inert effect of citrate and PVP against *E. coli* has also been reported by Ivask *et al.*^44^ Therefore, the antibacterial activity described above results from the AgNPs and not the capping agents as they mainly work as a stabilising coating.

#### 2.3.2. Bacterial growth curves

**Fig. 8:**
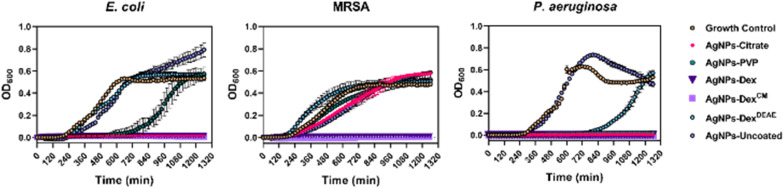
Growth curves of *E. coli*, MRSA and *P. aeruginosa* exposed to the AgNPs. The equivalent concentration of silver against *E. coli* and MRSA was 30 μg mL^−1^, and against *P. aeruginosa* was 7.5 μg mL^−1^.

The growth of the bacteria exposed to the AgNPs was monitored over 21 hours. Fig. 8 presents the optical density at 600 nm (OD_600_) of MRSA, *E. coli* and *P. aeruginosa* exposed to the AgNPs at equivalent silver concentrations. This was 30 μg mL^−1^ of silver for *E. coli* and MRSA, and 7.5 μg mL^−1^ for *P. aeruginosa*. The effect of the AgNPs on the growth rates, lag times, and asymptotic growth was also analysed by fitting the experimental data to the Gompertz model (Table S3 and Fig. S9†).

The results show the AgNPs-citrate, AgNPs-Dex and AgNPs-Dex^CM^ completely inhibited the growth of *E. coli*, with the AgNPs-PVP being able to prolong the lag phase for 10 hours. As expected, the bare AgNPs and AgNPs-Dex^DEAE^ did not decrease the *E. coli* growth rate.

Regarding MRSA, all the AgNPs decreased the growth rate (Table S3 and Fig. S9†), except the AgNPs-Dex^DEAE^, nonetheless just the AgNPs coated with Dex and Dex^CM^ inhibited the bacterial growth. These results agree with the MIC results, except for the AgNPs-Dex, which had a higher MIC (60 μg mL^−1^). The discrepancy probably results from the differences in the assay settings. While in the MIC test the bacteria were left in an incubator at 37 °C under static conditions, in the growth curves assay, the bacteria were left inside a microplate reader, also at 37 °C, but stirred every 20 min for 20 s before each OD_600_ reading. This helped to increase the oxygenation of the medium, and consequently increase the silver oxidation and release of Ag^+^ that exerts the toxic effect. The increased toxicity of AgNPs under aeration conditions has been previously reported by Xiu Z. *et al.*^18^ and explained by the aeration increasing the oxygen exposure and promoting the release of higher quantities of Ag^+^.

Regarding *P. aeruginosa*, the concentration of AgNPs tested (7.5 μg mL^−1^) was lower than the concentration tested against *E. coli* and MRSA (30 μg mL^−1^), as *P. aeruginosa* is more sensitive to the AgNPs. This effect was shown with the MIC and MBC results and corroborated by the growth curves, where AgNPs-uncoated and AgNPs-Dex^DEAE^ were the only nanoparticles that did not inhibit the bacterial growth. However, the AgNPs-Dex^DEAE^ prolonged the lag phase for about 13 hours.

Overall, the growth curves highlight the trend presented in the MIC and MBC results, *i.e.*, *P. aeruginosa* is more sensitive to AgNPs than *E. coli*, and *E. coli* is more sensitive to AgNPs than MRSA. This trend was verified for all the coated nanoparticles and is believed to result from structural differences between bacteria, and different mechanisms used to evade the toxic effect of the nanoparticles. Similar trends where the AgNPs present stronger activity against Gram-negative than Gram-positive have been previously reported and is believed to result from differences in the cell wall.^40,45–47^ Moreover, the strong activity of AgNPs against *P. aeruginosa*, a bacterium that has developed an increased resistance to antibiotics is believed to result from AgNPs enhancing the oxidative stress and interfering with the ability of the bacteria to form biofilms by decreasing their adhesion, motility, destroying the iron homeostasis, blocking aerobic and anaerobic respiration, and affecting the quorum sensing systems.^48,49^

#### 2.3.3. Biocidal activity against formed biofilm

The effect of AgNPs on the removal of biofilms was assessed against established biofilms of MRSA and *P. aeruginosa*. *E. coli* was not tested as the strain used in this work did not form strong biofilms.

The destruction of biofilms is challenging, as they present additional mechanisms to inactivate and surpass the antimicrobial agents. In the case of metal-containing salts and nanoparticles, the polysaccharides in the extracellular matrix of the biofilm can stop their diffusion *via* chelation, entrapment, or agglomeration.^50^ This reduces the activity of the nanoparticles or metallic ions, and can stimulate the appearance of antimicrobial resistance.^51^ Moreover, a considerable number of bacterial cells in the biofilms are in a stationary phase, making them less susceptible to antimicrobials agents that depend on the metabolism of the cells to exert their activity.^50–52^

The activity of nanoparticles against biofilms depends on the particle size, composition, charge and surface chemistry. All these factors affect the transport of the nanoparticles into the biofilms, and therefore the antimicrobial activity.^53^ In the case of AgNPs, it has been shown that smaller nanoparticles are more effective in removing biofilms, due to better penetration into the biofilms and greater silver ions dissolution.^54–57^

**Fig. 9:**
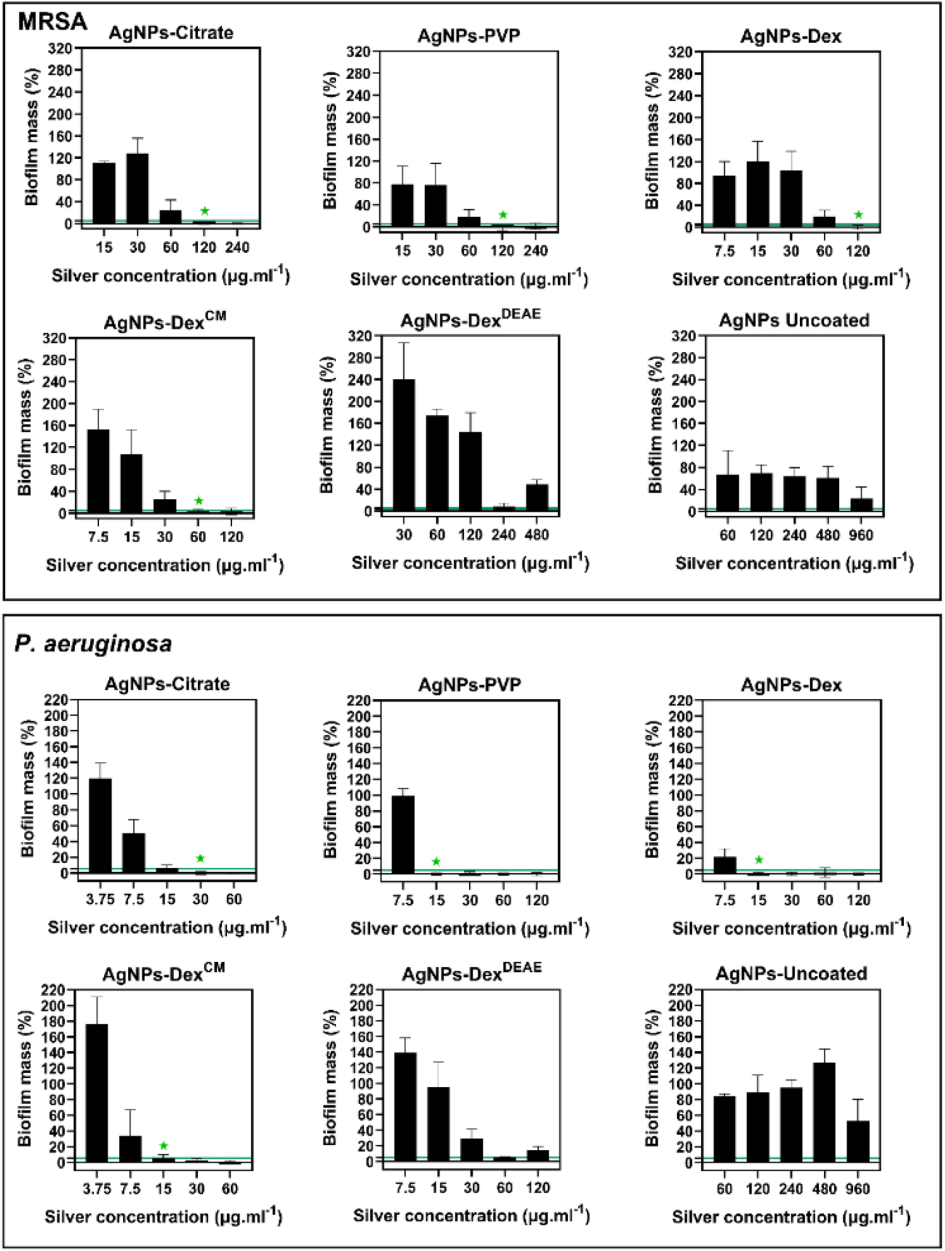
Remaining biofilm mass after treatment of the MRSA or *P. aeruginosa* biofilms with AgNPs at different equivalent concentrations of silver. The green line represents the minimum biofilm mass cut-off (≤5%) for the definition of the minimum biofilm eradication concentration (MBEC) and the green star marks the MBEC value.

Fig. 9 presents the percentage of biofilm mass left after treatment with different concentrations of AgNPs, and Table S4† the MBEC. The minimum concentration of silver that promoted the eradication of 95% or more of the MRSA biofilm was 120 μg mL^−1^ for the AgNPs coated with citrate, PVP and Dex, and 60 μg mL^−1^ for the AgNPs coated with AgNPs-Dex^CM^. Once again, the AgNPs-Dex^CM^ presented better activity against MRSA, which can be explained by its better stability and diffusion into the biofilm, as smaller nanoparticles tend to travel deeper in the biofilm. The AgNPs-Dex^DEAE^ and AgNPs-uncoated decreased the biofilm mass at high concentrations however, the remaining biofilm mass was above the MBEC threshold (<5%). Peulen *et al.*^57^ studied the diffusion of nanoparticles into biofilms composed of *P. fluorescens* and found that the diffusion decreased exponentially with the square of the solute radius, and the effective size of the biofilm pores ranged between 10 to 50 nm. Based on these findings, the poor activity of the AgNPs-Dex^DEAE^ and AgNPs-uncoated can be explained by their larger sizes. While the uncoated AgNPs presented a broad distribution of diameters (Fig. 3) due to their uncontrolled growth during synthesis, the AgNPs-Dex^DEAE^ formed micro-sized clusters in MHB-S (Fig. 6) due to the poor stability in the medium. Moreover, the uncoated AgNPs are more prone to agglomeration due to the lack of a stabilising agent. Due to these reasons, both nanoparticles had poor diffusion into the biofilm, which explains their lower bactericidal activity against formed biofilm.

As expectable, the *P. aeruginosa* biofilms were more sensitive to the AgNPs, with the AgNPs coated with PVP, Dex and Dex^CM^ presenting an MBEC of 15 μg mL^−1^, and the AgNPs coated with citrate of 30 μg mL^−1^. Once again, the AgNPs-Dex^DEAE^ and AgNPs-uncoated did not completely eradicate the biofilm due to their larger sizes and limited diffusion.

Interestingly, at some sub-lethal concentrations, mainly for the AgNPs coated with Dex^CM^ and Dex^DEAE^, the nanoparticles promoted an increased biofilm formation for both bacteria (Fig. 9). This effect has been previously reported by Yang *et al.*^58^ for *P. aeruginosa*, and is believed to be caused by sublethal concentrations of AgNPs stimulating biofilm formation *via* upregulation of the quorum sensing, lipopolysaccharide biosynthesis, antibiotic resistance genes, and the enhanced production of components of the biofilm matrix like sugars and proteins.

Overall, the data shows that the AgNPs activity against the biofilms is highly dependent on their size and stability, with the capping agent playing an essential role in keeping the antibacterial activity of the AgNPs.

## 3. Conclusion

In the present study the effect of five different capping agents (citrate, PVP, Dex, Dex^CM^ and Dex^DEAE^) on the physicochemical properties and antibacterial activity of the AgNPs was studied. The results showed that the capping agents are essential to prevent the nanoparticles growth over nanoscale dimensions and endow stability. The nanoparticles synthesised with capping agents presented diameters between 8 and 13 nm, while the uncoated AgNPs presented a larger average diameter (33 nm) due to the uncontrolled growth during synthesis. All the AgNPs, without exception, presented the typical crystalline structure of pure silver, without the presence of the silver oxide peaks. The DLS and FTIR analysis demonstrated that the coated AgNPs acquired the properties of the capping agent in terms of charge and spectral signatures, being more stable during storage than the bare AgNPs, most probably due to the repulsions forces and steric hindrance created by the capping agents.

The stability of the AgNPs in PBS and different media used to grow bacteria was also studied. Overall, the data showed that the AgNPs are highly sensitive to the composition of the media and buffer, with positively charged nanoparticles being extremely unstable in culture media. The higher the content of electrolytes and infusion or extracts in the media, the higher the disruption of the stability, with the nanoparticles stabilised through electrostatic repulsions being more easily destabilised.

The antibacterial activity of the AgNPs was tested against *E. coli*, MRSA and *P. aeruginosa*. The results showed that the antibacterial activity of the AgNPs was dependent on their stability and the interaction with the medium and the bacteria. The AgNPs coated with Dex and Dex^CM^ presented the strongest activity across all the antibacterial tests (MIC, MBC and MBEC) due to their better stability, smaller size, and increased interactions with the bacteria.

In conclusion, this work shows that the capping agents have a crucial role in maintaining the physicochemical properties and antibacterial activity of the AgNPs. From production to the final application, the capping agents protect the AgNPs from overgrowth and agglomeration. Importantly, the capping agent must be chosen taking into consideration the final application, as their charge and structure affect the affinity to the AgNPs and their stability. As shown in this work, small variations in the capping agents can considerably improve or deteriorate the antibacterial activity of the AgNPs.

## 4. Materials and methods

### 4.1. Materials

Silver nitrate (AgNO_3_, extra pure crystals, ≥99%), sodium borohydride (NaBH_4_, ≥99% pure), polyvinylpyrrolidone 40 kDa (PVP), trisodium citrate (citrate, C_6_H_5_Na_3_O_7_·2H_2_O, ≥99% pure), fluorescein isothiocyanate-dextran 40 kDa (Dex), fluorescein isothiocyanate-diethylaminoethyl-dextran 40 kDa (Dex^DEAE^), fluorescein isothiocyanate-carboxymethyl-dextran 40 kDa (Dex^CM^), dehydrated Mueller Hinton broth Sigma 70192 (MHB-S), Mueller Hinton agar Sigma 70191 (MHA-S), phosphate buffered saline (PBS) tablets, Triton™ X-100 ((C_2_H_4_O)_*n*_C_14_H_22_O) and TraceCERT® 1 ppm Silver ICP-MS standard in 2% nitric acid, were obtained from Sigma-Aldrich (Steinheim, Germany). Calcium dichloride dihydrate (CaCl_2_·2H_2_O, ≥99%), disodium carbonate (Na_2_CO_3_, ≥99.5%), tris buffer saline 10× solution (TBS), 99% ethanol, 70% nitric acid (HNO_3_, analytical grade), 37% hydrochloric acid (HCl, analytical grade), dehydrated Mueller Hinton Broth Oxoid CM0405B (MHB-O), dehydrated Nutrient Broth Oxoid CM0001B (NB), dehydrated Lennox Broth Base 12780-052 Thermo Fisher Scientific (LB) and crystal violet (C_25_H_30_N_3_Cl, pure), were obtained from Fisher Scientific (Loughborough, United Kingdom).

### 4.2. Methods

#### 4.2.1. Synthesis of the AgNPs

AgNPs were synthesised *via* a modified chemical reduction methodology adapted from Nau E. *et al.*^19^ Briefly, freshly prepared NaBH_4_ (40 mL, 0.01 M) was added dropwise (*ca.* 2 drops per s) at room temperature and under stirring (850 rpm) to AgNO_3_ (2 mL, 0.1 M) previously mixed with Milli-Q water (158 mL) and the capping agent (trisodium citrate, PVP, Dex, Dex^DEAE^ or Dex^CM^). The final capping agent concentration was 0.20, 0.38 and 0.13 mg mL^−1^ for trisodium citrate, PVP and all the dextrans, respectively. The concentration of trisodium citrate was chosen based on the work of Izak-Nau, E. *et al.*^19^ The concentrations of PVP and Dex^DEAE^ were selected based on preliminary studies, where AgNPs were synthesised with different concentrations of PVP (0.09 to 1.50 mg mL^−1^) and Dex^DEAE^ (0.02–0.38 mg mL^−1^) and then compared. After synthesis, AgNPs were filtered and then washed with Milli-Q water by centrifugation (5000*g* for 30 min) using Pierce™ Protein Concentrators PES with a 50k molecular weight cut-off membrane (Thermo Fisher Scientific, Germering, Germany). The particles were then resuspended in deionised water, and the silver concentration was determined by inductively coupled plasma mass spectrometry (ICP-MS).

#### 4.2.2. Characterization of the AgNPs

##### 4.2.2.1 Ultraviolet-visible (UV-vis) spectroscopy

AgNPs present size-dependent optical properties, making UV-vis analysis a simple and highly sensitive method to evaluate AgNPs formation, size, and stability.^59,60^ Briefly, AgNPs were diluted with Milli-Q water, and the UV-vis extinction spectra were recorded in a NanoDrop One spectrophotometer (Thermo Scientific, USA) between 190 and 850 nm. The UV-vis spectra were normalised between 300 and 600 nm for better comparison between the samples.

##### 4.2.2.2 Transmission electron microscopy (TEM)

AgNPs stock colloidal dispersions were diluted with Milli-Q water, and then 7 μL were added on top of a holey carbon film copper grid (Agar Scientific Ltd., UK) and left drying overnight before analysis on a JEM-2100 Plus transmission electron microscope (Jeol, Japan) using an operating voltage of 200 kV. The diameter of 300 particles was measured to estimate the average particle size and distribution using the ImageJ software (NIH, USA).

##### 4.2.2.3 Dynamic light scattering (DLS) analysis

DLS analysis was performed to determine the hydrodynamic size and zeta-potential of the AgNPs in Milli-Q water (pH 5.28) using a Zetasizer Nano ZS instrument (Malvern, UK). The hydrodynamic size data corresponds to an average of 30 runs, and the zeta potential to the calculated mean value with an average of 50 runs.

##### 4.2.2.4 Fourier transform infrared (FTIR) spectroscopy

The AgNPs were analysed by FTIR spectroscopy (Spectrum Two FTIR spectrometer, PerkinElmer, Uberlingen, Germany) after being washed. The samples were dried just before analysis until no water peaks were detectable. 32 scans were run for each sample between 500 and 4000 cm^−1^, with a resolution of 4 cm^−1^. The ATR (attenuated total reflectance) technique was used in all the measurements. The spectra were normalised for better comparison between samples.

##### 4.2.2.5 X-ray diffraction (XRD)

The crystalline structure of the AgNPs was analysed in the SmartLab SE X-ray diffraction system from Rigaku Co. Ltd. (Tokyo, Japan) with a Kβ filter for cupper (*λ* = 0.1392 nm). Samples were scanned with a *θ*/2*θ* scan axis. The scan range varied between 20° and 80°, and the scan mode and speed were 1D and 5° min^−1^, respectively.

##### 4.2.2.6 Thermogravimetric analysis (TGA)

Approximately 3 mg of dried AgNPs were heated in an open porcelain crucible from 30 to 850 °C, under a nitrogen atmosphere at a heating rate of 10 °C min^−1^ on a thermogravimetric analyser (TGA 4000, PerkinElmer). The capping agents were also analysed at the same conditions for comparison.

##### 4.2.2.7 Inductively coupled plasma mass spectrometry (ICP-MS) for silver concentration determination

AgNPs colloidal dispersions were digested with a fresh mixture of one part of 70% HNO_3_ and three parts of 37% HCl (v/v) to ensure the formation of soluble silver chloride complexes (AgCl_*x*_^(*x*−1)−^) instead of insoluble AgCl salts. All the digested samples presented a concentration of silver lower than 10 μg mL^−1^ and an HCl content higher than 10% (v/v). The samples were digested at room temperature in the dark for over 1 hour and then 7 to 14 μL of the digested samples were diluted with 1 mL of 2% HNO_3_ before analysis. A calibration curve was obtained for each independent ICP analysis with silver concentrations ranging between 3 μg L^−1^ to 800 μg L^−1^. The coefficient of determination of the standards calibration curve was always superior to 0.99.

#### 4.2.3. Stability analysis of the AgNPs

The stability of all the synthesised AgNPs was analysed after being stored at 4 °C for 9 months. Briefly, fresh and 9 months old AgNPs were analysed by UV-vis spectroscopy and pictures were taken to assess the sedimentation of AgNPs. The stability of the different AgNPs was also analysed in PBS, and different types of broths used for bacterial growth/maintenance (MHB-S, MHB-O, LB and NB). Briefly, 25 μL of pre-washed AgNPs were mixed with 250 μL of Milli-Q water (control), PBS or broth, and then analysed by UV-vis spectroscopy, before and after overnight incubation at 37 °C (approx. 20 hours). The AgNPs were also analysed under the microscope (Life Technologies EVOS FL, Invitrogen, USA) to detect the formation of micro-size clusters. Milli-Q water, PBS, MHB-S, MHB-O, LB and NB without AgNPs were used as the respective blanks. The UV-vis spectra were normalised between 300 and 600 nm, and zero was defined as *y* ≤ 0.043 to eliminate the effect of background noise.

#### 4.2.4. Assessment of the antibacterial activity

The antibacterial activity of the AgNPs was determined against *Escherichia coli* O157:H7 (*E. coli*), methicillin-resistant *Staphylococcus aureus* (MRSA) and *Pseudomonas aeruginosa* PA01 (*P. aeruginosa*). The *E. coli*, MRSA and *P. aeruginosa* isolates were obtained from the American Type Culture Collection (ATCC 43888), National Collection of Type Cultures (NCTC 12493) and Nottingham Trent University (NTUCC 876) collection, respectively.

##### 4.2.4.1 Inoculum preparation

The bacterial isolates were streaked onto MHA-S plates and incubated at 37 °C for 18 to 20 hours. For each isolate, three to four isolated colonies of the same morphological appearance were transferred into a tube containing 5 mL of MHB-S and then incubated for 18–20 hours in a shaker at 35 °C and 225 rpm. Just before exposure of the bacteria to the AgNPs, overnight cultures were diluted to 1 × 10^6^ CFU mL^−1^.

##### 4.2.4.2 Minimum inhibitory concentration (MIC) and minimum bactericidal concentration (MBC)

The MIC was determined using the broth microdilution method adapted from Wiegand I. *et al.*,^61^ with some modifications. Briefly, the different AgNPs were serially diluted in a 96-wells microplate with MHB-S. Then 50 μL of *E. coli*, MRSA or *P. aeruginosa*, previously diluted with MHB-S to 1 × 10^6^ CFU mL^−1^, were added to each well. The concentrations of the AgNPs ranged between 480 and 0.47 μg mL^−1^. The final bacterial inoculum density was approximately 5 × 10^5^ CFU mL^−1^. Growth and sterility controls of the media and AgNPs were included in all the plates. The microplates were incubated at 37 °C for 18 to 20 h and then read visually by observing the presence or absence of turbidity. The MIC was defined as the lowest concentration that inhibited the visible growth of the bacteria in all the replicate wells. The MBC was determined after reading the MIC and consisted of plating 10 μL of the wells without visible turbidity onto MHA plates. The agar plates were incubated at 37 °C for 24 hours and then read visually. The lowest dilution without macroscopic bacterial growth was defined as the MBC. The experiment was repeated three times, with three replicates per repetition.

##### 4.2.4.3 Growth curves

Bacterial isolates were grown for 18–20 hours and then diluted to 1 × 10^6^ CFU mL^−1^ with MHB-S. After that, 50 μL of the diluted bacterial isolates were added to the wells of a 96-well microplate containing 50 μL of AgNPs in MHB-S. The concentration of the AgNPs against *E. coli* and MRSA was 30 μg mL^−1^ and against *P. aeruginosa* 7.5 μg mL^−1^. The bacterial growth was analysed in a microplate reader (Cytation 3, BioTek, Vermont, USA) over 21 hours. The microplate was kept at 37 °C under mainly static conditions, and the optical density was measured every 20 minutes at 600 nm. Before each measurement, the microplate was gently stirred in orbital movements for 20 s. The experiment was carried out in triplicate, and untreated samples were used as growth controls. Sterility controls of the media and AgNPs were also included in the experiment and used as blanks. To prevent condensation on the lid and the “edge effect” during incubation, the outer wells of the microplate were filled with PBS, and the lid was treated with 0.05% Triton X-100 in 20% ethanol as described by Brewster J.^62^ with some minor modifications. Briefly, 5 mL of 0.05% Triton X-100 in 20% ethanol, pre-filtered with a 0.2 μm syringe-tip filter (Fisherbrand™, Loughborough, United Kingdom), was poured on the microplate lid and tilted several times to ensure even coverage of the inner surface. The lid was then left for about 15 min inside the biosafety cabinet to prevent any contamination. After that, the Triton X-100 in 20% ethanol was poured off, and the lid was shaken to remove most of the liquid. Finally, the lid was leaned against a vertical surface inside the biosafety cabinet and allowed to air-dry.

The bacterial growth curves were fitted with the Gompertz model^63,64^ according to the following mathematical equation:
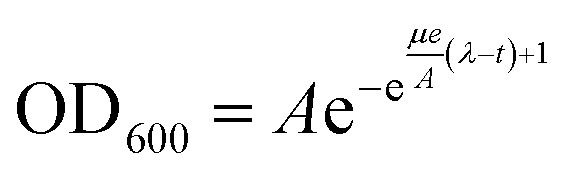
where, *λ* represents the lag time (min), *μ* the maximum growth rate (min^−1^) and *A* the asymptotic growth, as represented in Fig. S10.†

##### 4.2.4.4 Minimum biofilm eradication concentration (MBEC)

The MBEC determination method was adapted from Ivanova A. *et al.*^65^ with some modifications. Briefly, bacterial isolates of MRSA and *P. aeruginosa* were grown for 18–20 hours and then diluted to 1 × 10^6^ CFU mL^−1^ with MHB-S. Then 100 μL of bacteria were added to the wells of a 96-well microplate. The plates were then incubated for 24 hours at 37 °C under static conditions to promote biofilm formation. After incubation, the planktonic cells were removed by gently washing the wells two times with 150 μL of PBS. Between washes, the microplate was shaken at 100 rpm for 5 min. After washing, 120 μL of AgNPs at concentrations between 3.5 and 960 μg mL^−1^, previously diluted with MHB-S, were added to the wells and then the microplate was incubated for 24 hours at 37 °C under static conditions. After incubation, the non-adhered bacterial cells were removed by gently washing the wells two times with 150 μL of sterile PBS (between washes the microplate was shaken at 100 rpm for 5 min). After that, the biofilms were fixated at 60 °C for 1 hour and then stained with 150 μL crystal violet (0.1% w/v) for 1 hour at room temperature. After staining, the wells were washed three times with 150 μL of sterile PBS. Between washes, the microplate was shaken at 100 rpm for 5 min. The crystal violet was then dissolved with 200 μL per well of 30% v/v acetic acid. 100 μL of the dissolved crystal violet was then transferred into a 96-well microplate, and the absorbance read at 595 nm. The experiment was carried out in triplicate, and untreated biofilms were used as positive controls. Sterility controls were also carried out throughout the whole experiment and used as blank.

## Author contributions

Ana M. Ferreira: writing-original draft, writing-review & editing, conceptualization, data curation, methodology, validation, formal analysis, visualisation, investigation. Anna Vikulina: formal analysis, methodology, writing-review & editing. Michael Loughlin: supervision, methodology, writing-review & editing. Dmitry Volodkin: supervision, conceptualization, validation, methodology, writing-review & editing, project administration, resources, funding acquisition.

## Conflicts of interest

There are no conflicts to declare.

## Supplementary Material

RA-013-D3RA00917C-s001
