# Supplementary material for: How similar is the antibacterial activity of silver nanoparticles coated with different capping agents?
Source: RSC Adv. 2023 Apr 3;13(16):10542–55. doi: 10.1039/d3ra00917c (PMC10068916; doi:10.1039/d3ra00917c)
Supplement: RA-013-D3RA00917C-s001 [file RA-013-D3RA00917C-s001.pdf]

Electronic Supplementary Material (ESI) for RSC Advances.
This journal is © The Royal Society of Chemistry 2023

Supplementary information

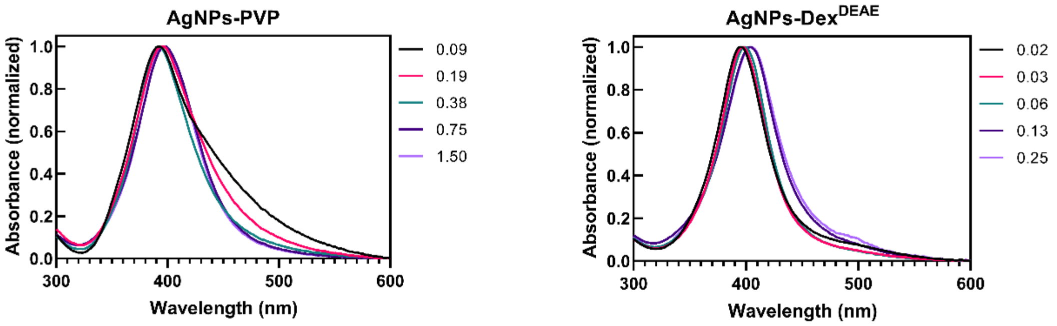

Figure S1. UV-vis spectra of AgNPs synthesised with different concentrations of PVP (right) and Dex^{DEAE} (left). Labels correspond to the final concentration of the capping agent in mg.ml^{-1}.

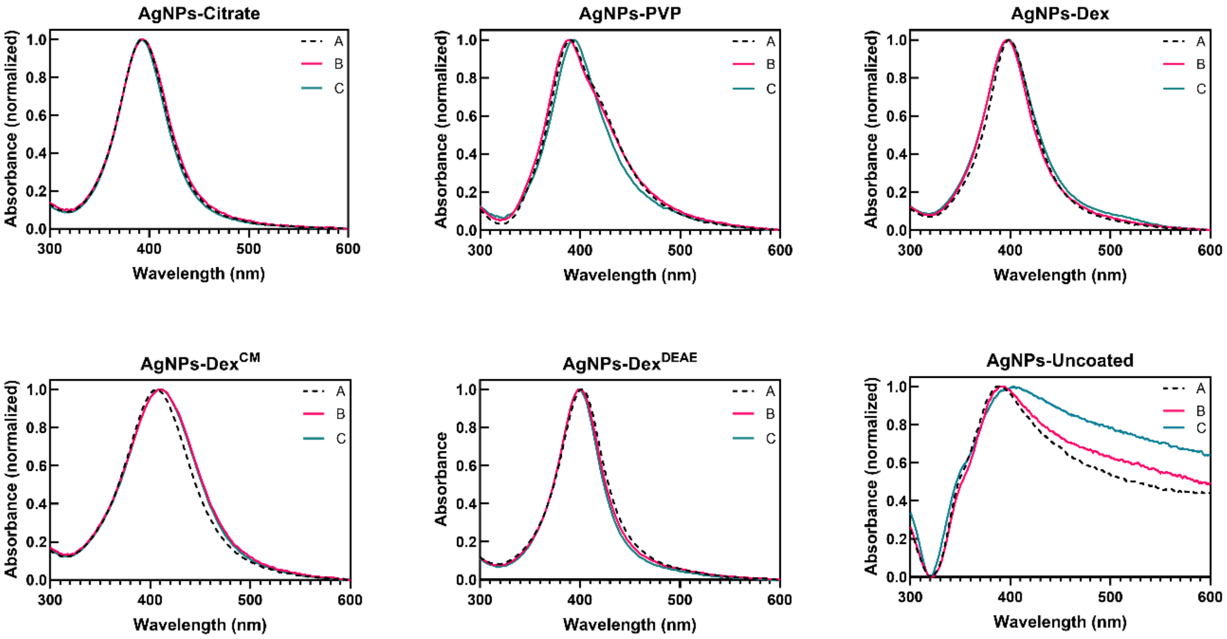

Figure S2. UV-vis spectra of independent replicates of AgNPs. The different replicates are labelled with different letters (A, B or C). The replicates were prepared on different days with fresh reagent solutions.

Table S1. Maximum absorbance wavelength (λmax) of the AgNPs after washing.

AgNPs	λmax (nm)
**AgNPs-Citrate**	389.0
**AgNPs-PVP**	394.5

**AgNPs-Dex**	398.0
**AgNPs-DexCM**	398.0
**AgNPs-DexDEAE**	406.0
**AgNPs-Uncoated**	388.5

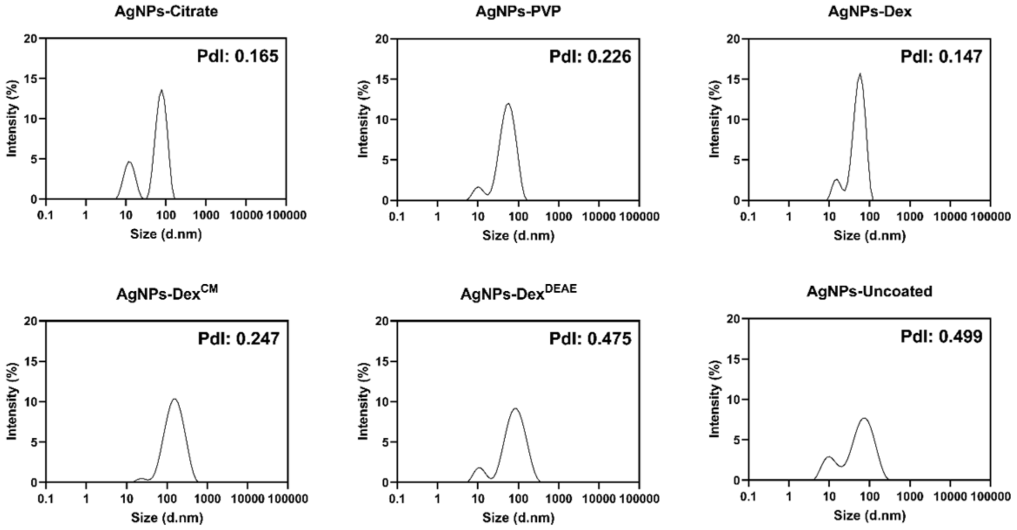

Figure S3. Size distribution by intensity determined by DLS of the AgNPs and corresponding polydispersity index (PdI).

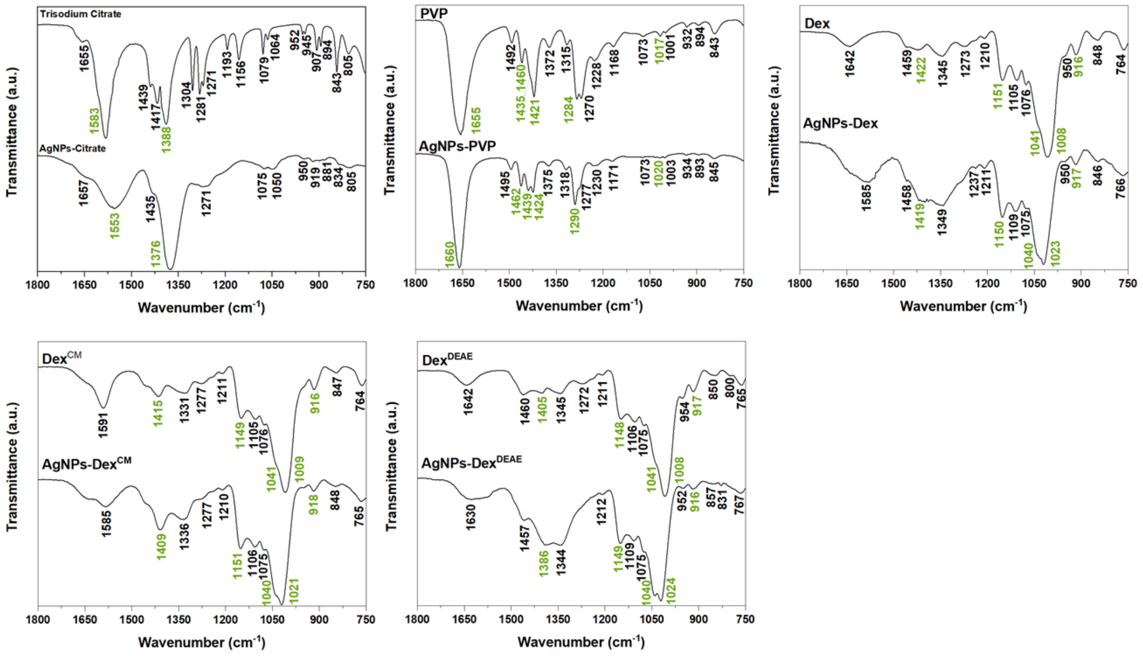

Figure S4. FTIR spectra of the AgNPs and respective capping agents.

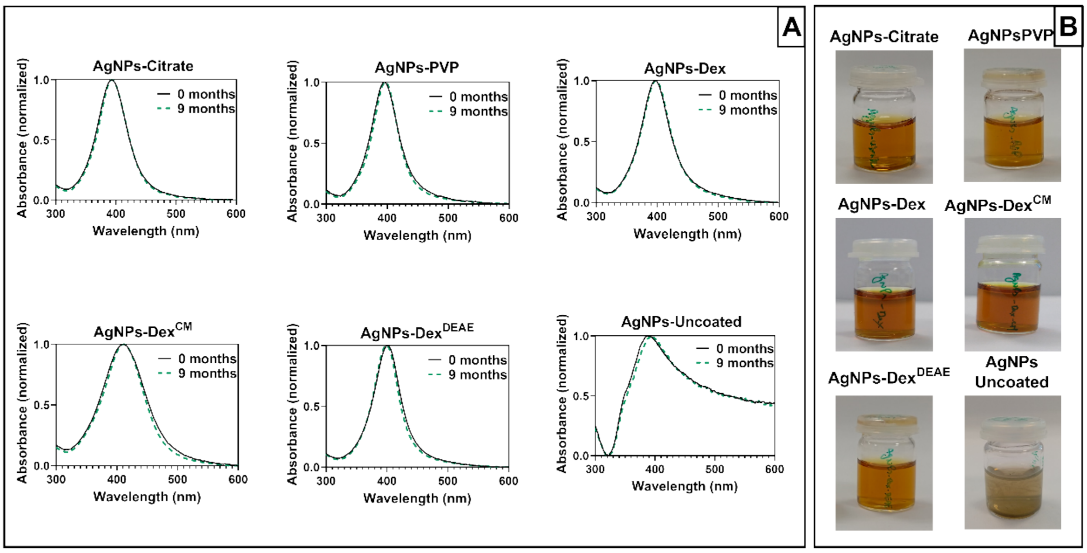

Figure S5. UV-vis spectra of the AgNPs after synthesis (0 months) and after being stored for 9 months (9 months) (A), and appearance of the colloidal dispersions after storage for 9 months (B).

Table S2. Composition of PBS buffer, MHB-O, MHB-S, LB and NB media. Values in brackets represent the concentration of the salt.

	**Salts ($g.L^{-1}$)**	**Dehydrated infusion from beef ($g.L^{-1}$)**	**Meat extract ($g.L^{-1}$)**	**Casein hydrolysate ($g.L^{-1}$)**	**Starch ($g.L^{-1}$)**	**Yeast extract ($g.L^{-1}$)**	**Peptone**
**PBS**	NaCl (8)<br>Na_2HPO_4/KH_2PO_4 (1)<br>KCl (10)		-	-	-	-	-
**MHB-O**	-	300	-	17.5	1.5	-	-
**MHB-S**	-	-	2	17.5	1.5	-	-
**LB**	NaCl (5)	-	-	-	-	5	10
**NB**	NaCl (5)	-	1	-	-	2	5

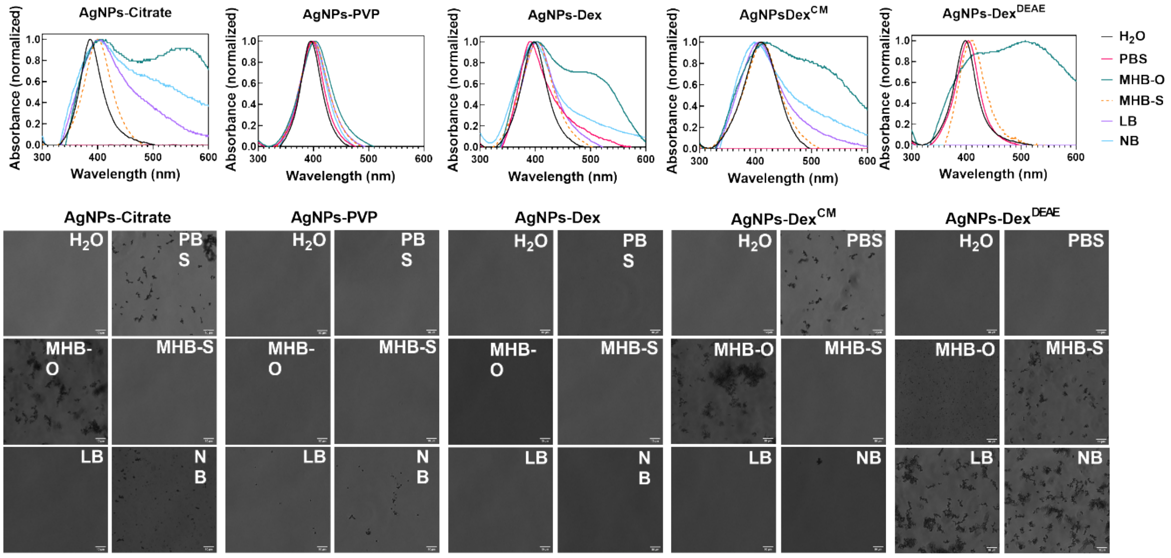

Figure S6. UV-vis spectra and respective transmittance images of the AgNPs in deionised water (H_2O), PBS and different broths used for bacterial growth: MHB-O, MHB-S, LB and NB.

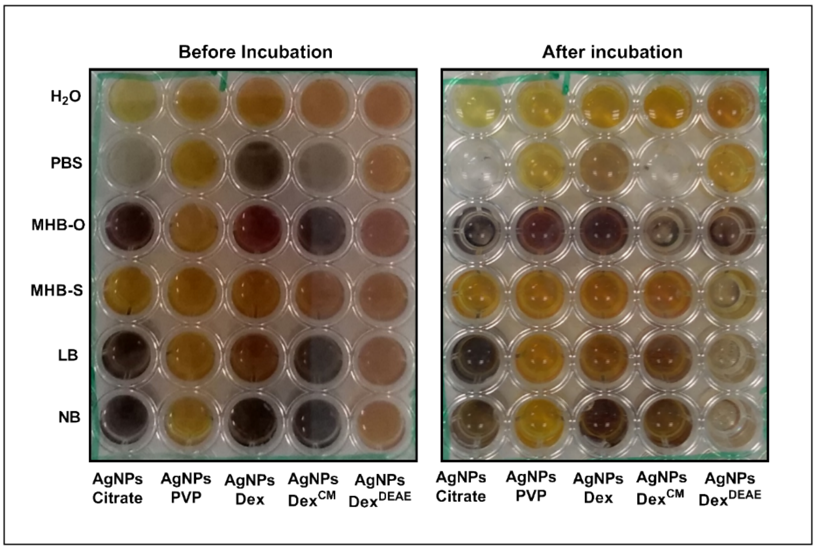

Figure S7. AgNPs in deionised water (H_2O), PBS and different broths used for bacterial growth: MHB-O, MHB-S, LB and NB, before and after overnight incubation at 37°C.

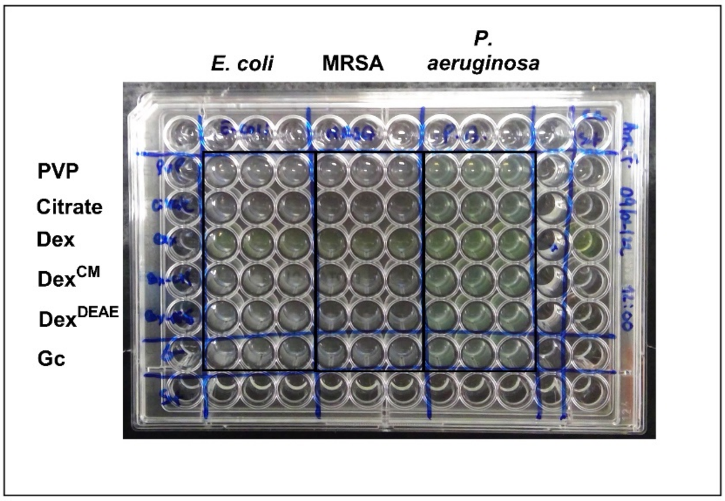

Figure S8. Microplate after incubation at 37°C for 20 hours with E. coli, MRSA and P, aeruginosa exposed to Citrate, PVP, Dex, Dex^{CM} and Dex^{DEAE}. The concentrations tested correspond to the theoretical amount of capping agent in a colloidal dispersion of AgNPs with a concentration of silver equal to 120 $\mu g.ml^{-1}$ (AgNPs-Dex and AgNPs-Dex^{CM}), 240 $\mu g.ml^{-1}$ (AgNPs-Citrate and AgNPs-Dex^{DEAE}), and 480 $\mu g.ml^{-1}$ (AgNPs-PVP). The concentrations tested are above the MBC. The growth control row (no capping agent) is labelled as Gc.

Table S3. Growth rates (μ), lag times (λ), and asymptotic growth (A) of the studied AgNPs against E. coli, MRSA and P. aeruginosa and regression coefficients (R^2).

Bacteria	AgNPs	λ (min)	μ (min^{-1})	A	R^2
	Growth Control	257	$1.6 \cdot 10^{-3}$	0.535	0.992
	AgNPs-Citrate	-	-	-	-
	AgNPs-PVP	737	$1.4 \cdot 10^{-3}$	0.713	0.992
E. Coli	AgNPs-Dex	-	-	-	-
	AgNPs-Dex^{CM}	-	-	-	-
	AgNPs-Dex^{DEAE}	261	$1.3 \cdot 10^{-3}$	0.588	0.996
	AgNPs-Uncoated	323	$1.3 \cdot 10^{-3}$	0.759	0.994
	Growth Control	241	$1.2 \cdot 10^{-3}$	0.487	0.998
	AgNPs-Citrate	267	$7.9 \cdot 10^{-4}$	0.663	0.999
	AgNPs-PVP	204	$7.9 \cdot 10^{-4}$	0.607	0.997
**MRSA**	AgNPs-Dex	-	-	-	-
	AgNPs-Dex^{CM}	-	-	-	-
	AgNPs-Dex^{DEAE}	162	$1.3 \cdot 10^{-3}$	0.497	0.999
	AgNPs-Uncoated	326	$8.9 \cdot 10^{-4}$	0.568	0.996
	Growth Control	345	$2.0 \cdot 10^{-3}$	0.711	0.978
	AgNPs-Citrate	-	-	-	-

P. aeruginosa	AgNPs-PVP	-	-	-	-
	AgNPs-Dex	-	-	-	-
	AgNPs-Dex^{CM}	-	-	-	-
	AgNPs-Dex^{DEAE}	986	$2.2\cdot10^{-3}$	2.455	0.994
	AgNPs-Uncoated	371	$2.1\cdot10^{-3}$	0.936	0.999

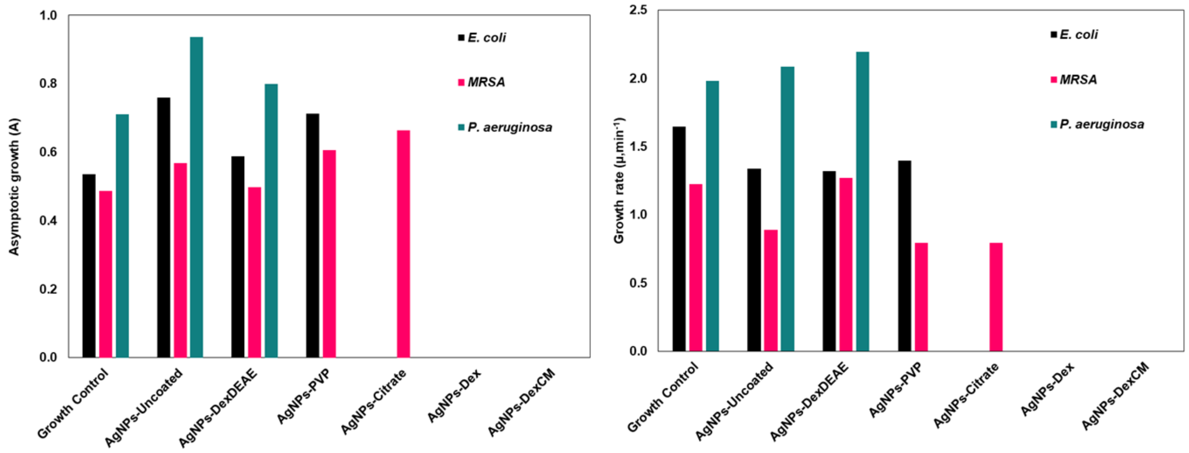

Figure S9. The influence of the capping agent on the growth rate µ and asymptotic growth A of AgNPs against E. coli, MRSA and P. *aeruginosa*.

Table S4. MBEC of the AgNPs against MRSA and *P. aeruginosa* biofilms. The concentration represents the content of silver in the AgNPs. Specify medium used.

**MBEC (µg.ml^{-1})**	**MRSA**	***P. aeruginosa***
AgNPs-Citrate	120	30
AgNPs-PVP	120	15
AgNPs-Dex	120	15
AgNPs-Dex^{CM}	60	15
AgNPs-Dex^{DEAE}	>480	>120
AgNPs-Uncoated	>960	>960

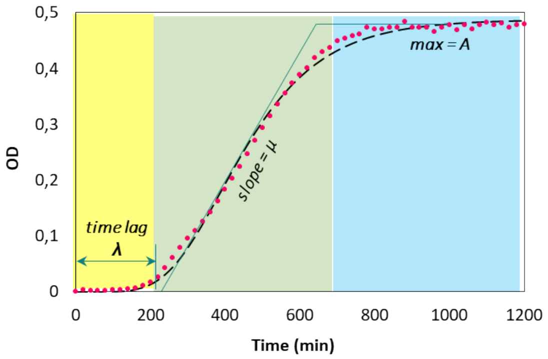

Figure S10. Typical growth curve of MRSA in the absence of AgNPs. Rosa points are the experimental data. Black dotted curve represents the fitting to Gompertz model. The growth curve has three growth phases: lag phase (yellow), log phase (green), and stationary phase (blue).
